# Oral human papillomavirus (HPV) infection in men who have sex with men: prevalence and lack of anogenital concordance

**DOI:** 10.1136/sextrans-2014-051955

**Published:** 2015-04-17

**Authors:** Eleanor M King, Richard Gilson, Simon Beddows, Kate Soldan, Kavita Panwar, Carmel Young, Mark Jit, W John Edmunds, Pam Sonnenberg

**Affiliations:** 1 Research Department of Infection and Population Health, University College London, London, UK; 2 The Mortimer Market Centre, Central and North West London NHS Foundation Trust, London, UK; 3 Virus Reference Department, Public Health England, London, UK; 4 Centre for Communicable Disease Surveillance and Control (CIDSC), Public Health England, London, UK; 5 Modelling and Economics Unit, Public Health England, London, UK; 6 Department of Infectious Disease Epidemiology, London School of Hygiene & Tropical Medicine, London, UK

**Keywords:** HPV, ORAL CAVITY, SEXUAL HEALTH, VACCINATION, GAY MEN

## Abstract

**Objectives:**

To estimate the prevalence of oral detectable human papillomavirus (HPV) DNA in HIV-negative men who have sex with men (MSM) attending a sexual health clinic in London and concordance with anogenital HPV infection. Such data are important to improve our understanding of the epidemiology of oral HPV and the potential use of vaccines to prevent oropharyngeal cancers.

**Methods:**

Paired oral rinse samples and anogenital samples were available from 151 HIV-negative MSM within a larger cross-sectional survey. All samples were tested in parallel for 21 types of HPV DNA using an in-house assay.

**Results:**

The median age of participants was 30 (IQR 25–35). The prevalence of any oral HPV and of high-risk HPV (HR-HPV) was 13.7% (n=21; 95% CI 8.7 to 20.2) and 5.9% (n=9; 95% CI 2.7 to 10.9) compared with 64.9% (n=98; 95% CI 56.7 to 72.5) and 34.4% (n=52; 95% CI 26.9 to 42.6) in any anogenital sample, respectively. The prevalence of types prevented by the bivalent (HPV16/18), quadrivalent (HPV6/11/16/18) and nonavalent (HPV6/11/16/18/31/33/45/52/58) vaccines was 1.3% (95% CI 0.2 to 4.7), 2.6% (95% CI 0.7 to 6.6) and 4.6% (95% CI 1.9 to 9.3), respectively. There was no concordance between HPV genotypes detected in oral and anogenital sites.

**Conclusions:**

HR-HPV DNA, including HPV 16/18, was detected in oral specimens from HIV-negative MSM attending sexual health clinics, suggesting a potential role for vaccination, but is far less common than anogenital infection. How this relates to the risk and natural history of HPV-related head and neck cancers warrants further study. Lack of concordance with anogenital infection also suggests that oral HPV infection should be considered separately when estimating potential vaccine impact.

## Introduction

Human papillomavirus (HPV) infection is associated with cancers at a number of sites in the head and neck, most importantly oropharyngeal cancer, which has been rising in incidence in recent decades.^1^ The global estimate of oral HPV prevalence in men was 5%^2^ and estimates in men who have sex with men (MSM) outside the UK range from 3% to 57%,^3^ ^4^ yet few studies have compared oral HPV prevalence in heterosexual and MSM populations.^3^ Furthermore, the natural history of oral HPV infection and its role in the development of head and neck cancers is not well understood.^5^ HPV infection and related disease in MSM is of particular interest, given the potential for prevention by vaccination.^6^

Estimating the potential benefit of vaccinating MSM requires knowledge of the epidemiology of oral HPV infection. To date, only three studies involving a total of 877 HIV-negative MSM have been conducted to estimate oral high-risk HPV (HR-HPV) prevalence, and results are not consistent (2%,^7^ 9%,^4^ 17%^8^). Likewise, the relationship between oral and anogenital HPV infection in MSM is not well understood.

Most transmission models of HPV infection used to inform vaccine programme design assume that infection occurs through anogenital contact, ignoring the role of oral–anogenital contact. Indeed, estimates of concordance between oral and anogenital HPV infection not only have implications for vaccine assessment but also for understanding the key epidemic characteristics such as route of acquisition, whether anogenital infections are acquired simultaneously or via separate risk acts/partnerships, or whether men transfer infections from one anatomical site to another (auto-inoculation).

We describe age-specific oral HPV prevalence and the concordance between detection of type-specific HPV DNA in oral and anogenital specimens from HIV-negative MSM attending a sexual health clinic in central London.

## Methods

Study methods, including sample size calculations, have been reported elsewhere.^9^ In brief, from October 2010 to July 2012, a cross-sectional study was conducted to examine the risk factors and prevalence of HPV DNA in MSM attending the sexual health clinic at the Mortimer Market Centre (MMC), London. Consecutive men aged 16–40 years, who reported anal or oral sex with another man in the last 5 years, were invited to participate. Participants provided written informed consent, completed a computer-assisted self-interview questionnaire and anogenital specimens (first-void urine, intra-anal swab and external genital swab (glans penis/coronal sulcus/penile shaft/scrotum/perianal area)) were collected by the study nurse using a standardised protocol.

Participants who enrolled in the final five months of the study were also invited to provide an oral specimen involving a 30 s gargle/rinse with 15 mL of Scope mouthwash according to a published protocol.^3^ Oral samples were refrigerated immediately and processed the same day. To process, the rinse was centrifuged at 3200 rpm for 15 min at 4°C and, after the supernatant was discarded, the pellet was resuspended in 20 mL of cold phosphate buffered saline (PBS) (4°C). The centrifugation/resuspension was repeated twice and the final pellet was resuspended in 1.2 mL of PBS with repeat pipetting and vortexing to ensure a homogeneous sample. Samples were stored at −20°C until shipment to the laboratory on dry ice at the end of the study. Specimens were removed for batch processing wherein all available specimens from each individual were processed for DNA extraction and HPV testing in the same run. Nucleic acid extraction and PCR amplification, HPV genotyping and determination of specimen integrity methods have been previously described.^10^ HPV genotypes 16/18/31/33/35/39/45/51/52/56/58/59/68 were considered HR-HPV types.

Here we report HPV prevalence and demographic and behavioural characteristics among participating MSM with both oral and anogenital specimens that were adequate for PCR-based HPV detection, for bivalent (HPV16/18), quadrivalent (HPV6/11/16/18) and nonavalent (HPV6/11/16/18/31/33/45/52/58) vaccine-preventable types together and individually, and for selected other HPV types: 35/39/51/56/59/68/26/53/66/70/73/82. CIs (95%) around prevalence estimates were determined by the Clopper–Pearson (exact) method.

## Results

There were 522 participants in the full study (n=522) and 177 gave an oral specimen. Participant and specimen numbers are shown in online supplementary figure S1. The study population was slightly younger than MSM attending MMC during the recruitment period (data not shown) and were more likely to be a new patient at the clinic than those who declined to participate; 163/173 (94%) HIV-negative MSM provided oral specimens, 153/173 (88%) were adequate for HPV testing and 151/173 (87%) had at least one matching anogenital specimen (ie, urine, anal swab or external genital swab). Also, 127/151 (84%) had an anal swab, 135/151 (89%) had an external genital swab and 143 (95%) had a urine specimen.

Online supplementary table S1 displays characteristics of substudy participants. The median age of respondents with adequate paired specimens was 30 (IQR 25–35), 75% were of white ethnicity and 64% had >30 lifetime sexual partners.

Nine MSM had HR-HPV DNA detected in their oral specimens, and three had LR-HPV. Table 1 shows the oral prevalence of any HPV was 14% (95% CI 9% to 21%), HR-HPV was 6% (95% CI 3% to 11%) and of HPV16 was 0.7% (95% CI 0% to 4%). Prevalence estimates of bivalent, quadrivalent and nonavalent vaccine-preventable types were 1%, 3% and 5%, respectively. By comparison, the anogenital prevalence of any HPV was 65% (n=98; 95% CI 57 to 73) and HR-HPV was 34% (n=52; 95% CI 27 to 43). None of the 151 pairs had the same HPV type detected in both oral and anogenital samples; 14 (9%) pairs had any HPV detected in both oral and anogenital sites (see online supplementary table S2).

**Table 1:** Human papillomavirus (HPV) DNA prevalence in oral cavity of men who have sex with men

			N=151
	n	Per cent	95% CI
Any HPV type*	21	13.9	8.8 to 20.5
Multiple types (two or more)	1	0.7	0.0 to 3.6
Multiple HR types† (two or more)	0	0.0	0.0 to 2.4
Any bivalent vaccine types‡	2	1.3	0.2 to 4.7
Any quadrivalent vaccine types‡	4	2.6	0.7 to 6.6
Any nonavalent vaccine types‡	7	4.6	1.9 to 9.3
Possible high-risk types§	0	0.0	0.0 to 2.4
High-risk HPV types (HR-HPV)†	9	6.0	2.8 to 11.0
HPV16	1	0.7	0.0 to 3.6
HPV18	1	0.7	0.0 to 3.6
HPV31	0	0.0	0.0 to 2.4
HPV33	1	0.7	0.0 to 3.6
HPV35	0	0.0	0.0 to 2.4
HPV39	0	0.0	0.0 to 2.4
HPV45	1	0.7	0.0 to 3.6
HPV51	1	0.7	0.0 to 3.6
HPV52	1	0.7	0.0 to 3.6
HPV56	3	2.0	0.4 to 5.7
HPV58	0	0.0	0.0 to 2.4
HPV59	0	0.0	0.0 to 2.4
HPV68	0	0.0	0.0 to 2.4
Low-risk HPV types			
6 and/or 11¶	3	2.0	0.4 to 5.7
6¶	3	2.0	0.4 to 5.7
11	0	0.0	0.0 to 2.4

*Sample reacted to the universal probe for HPV DNA.

†HR types 16/18/31/33/35/39/45/51/52/56/58/59/68 classified according to the International Agency for Research on Cancer monograph carcinogenic or probably carcinogenic.

‡Vaccine types: HPV16/18 are bivalent, HPV6/11/16/18 are quadrivalent and HPV6/11/16/18/31/33/45/52/58 are nonavalent.

§Possible high-risk types: 26/53/66/70/73/82.

¶One with co-infection with HPV18.

## Discussion

Our observed prevalence of oral HR-HPV of 6% was higher than that of the global estimate in healthy men and women (3.5%; 95% CI 3.0% to 4.1%) yet the CIs overlap.^2^ Our results lie within the range of estimates for HIV-negative MSM.^4^ ^7^ ^8^ Although MSM are at high risk of anogenital HPV-related disease, none of the men with an oral HPV infection had the same genotype of HPV infection at any anogenital site. The prevalence of any HPV type was also higher in anogenital than oral samples.

Differences in estimates of oral prevalence between studies could be due to both technical and population differences. Methods of oral specimen collection, processing, storage, DNA extraction and test sensitivity and specificity differ across studies. We followed the oral rinse collection protocol from the Human Papillomavirus Infection in Men (HIM) study that is thought to yield more HPV DNA from the entire oral cavity than oral swabs and used a similar processing method.^w1^

In the longitudinal Multicenter AIDS Cohort (MAC) study, HPV, particularly HR-HPV, in MSM was more likely to persist at the anus than the oral cavity, while heterosexual men were as likely to have persistent oral HPV as MSM, but less likely to have persistent anal HPV.^8^ In the HIM study in which only 3.5% were MSM, HPV persistence was similar at oral and anogenital sites but the incidence was lower in the oral cavity compared with anogenital sites.^w2^ In a cross-sectional assessment, persistent infections cannot be differentiated from those that are transient, and we did not measure oral cavity symptoms, but our lower oral prevalence as compared to anogenital prevalence may be due to both a lower incidence at oral sites and a lower proportion of persistent infections. While we have grouped HR-HPV types, defined by their oncogenic potential at the cervix, there is only evidence for HPV16 being associated with head-and-neck cancers.^w3^

Our finding of a lack of concordance between oral and anogenital specimens is similar to those in HIV-positive men reported by Videla *et al* (predominantly MSM)^w4^ and Parisi *et al*.^w5^ However, Edelstein *et al* found that 15/17 men (predominantly heterosexual) had the same type detected at oral and anogenital sites.^w6^ Lower prevalence in the oral cavity and the lack of concordance with anogenital infection suggest that oral HPV infections are acquired independently of anogenital infections, or that they are less likely to reactivate from latency than anogenital infections, and would also suggest that auto-inoculation does not commonly occur. Differences in sensitivity of the detection methods may also have contributed to some disparity in HPV detection between anatomical sites but is unlikely to account for the magnitude of the difference observed.

We have previously demonstrated that our study population is at greater risk of STI acquisition than MSM attending other SHCs in England and MSM who attend SHCs in the general population of Britain.^9^ If oral HPV infection is a function of partner change, the estimates of oral HPV prevalence in our MSM population may be inflated compared with MSM attending SHCs more generally in Britain.

The sample size of this substudy was too small to accurately estimate oral–anogenital concordance (if true population prevalence is below 6%) but could estimate oral prevalence within 5% if the true population prevalence was 13%.

HPV vaccines can prevent oral HPV infections,^w7^ but whether they are effective against HPV-related head and neck cancers has yet to be shown. The lack of concordance between oral and anogenital specimens suggests that the additional potential benefit of HPV vaccination for preventing oral HPV-related disease should be considered in epidemiological and natural history models, in addition to anogenital HPV infection. Further studies, including meta-analyses, are needed to elucidate the epidemiology and natural history of oral HPV infection.

## Supplementary Material

Web figure

Web references

Web table 1

Web table 2
