# Supplementary material for: Oral human papillomavirus (HPV) infection in men who have sex with men: prevalence and lack of anogenital concordance
Source: Sex Transm Infect. 2015 Apr 17;91(4):284–6. doi: 10.1136/sextrans-2014-051955 (PMC4453633; doi:10.1136/sextrans-2014-051955)
Supplement: Web figure [file sextrans-2014-051955-s1.pdf]

Supplementary figure 1. Flowchart of participants and available specimens for the estimation of HPV DNA prevalence in MSM

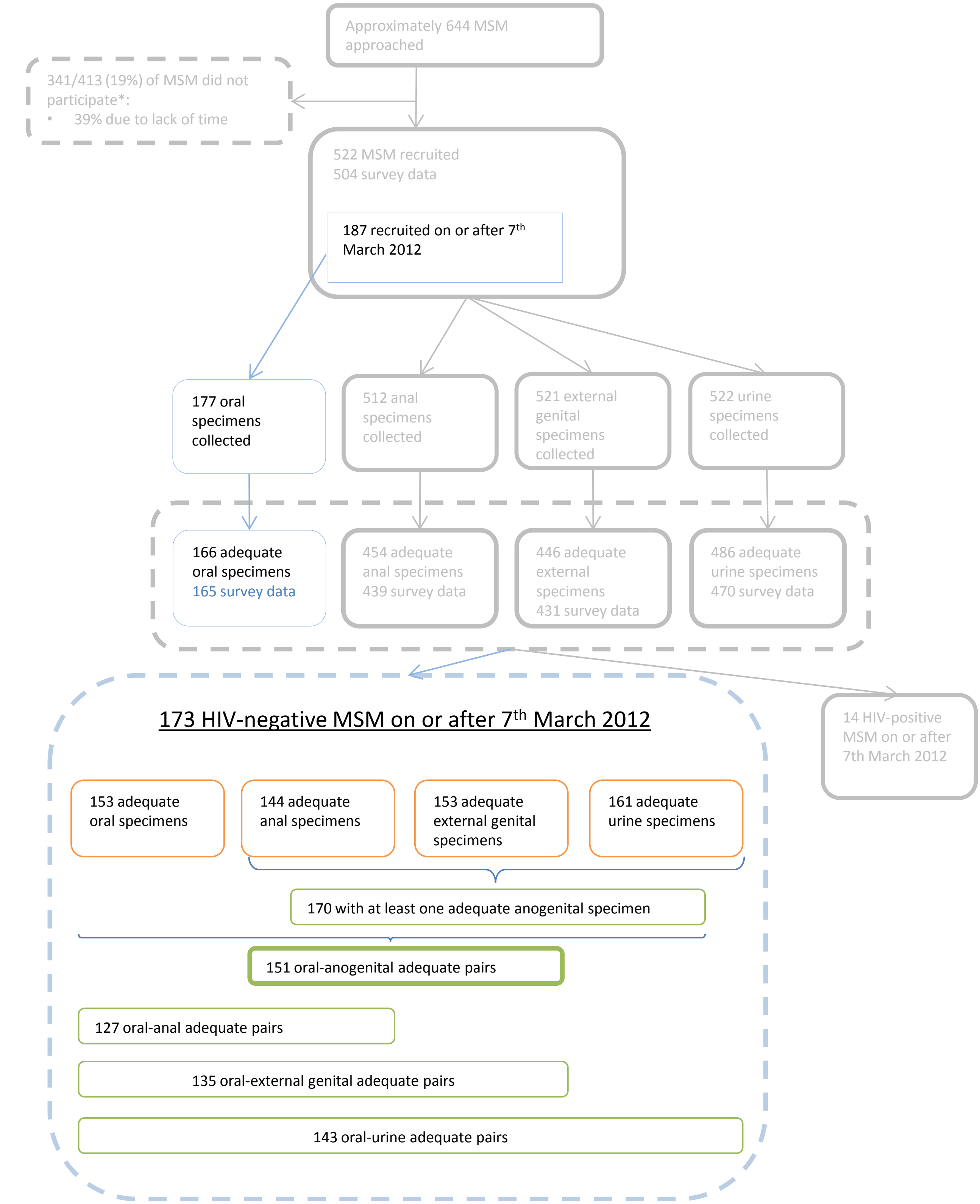

*Incomplete data due to interrupted completion of screening log
