# Supplementary material for: Oral human papillomavirus (HPV) infection in men who have sex with men: prevalence and lack of anogenital concordance
Source: Sex Transm Infect. 2015 Apr 17;91(4):284–6. doi: 10.1136/sextrans-2014-051955 (PMC4453633; doi:10.1136/sextrans-2014-051955)
Supplement: Web references [file sextrans-2014-051955-s2.pdf]

Web references

w1 Steinau M, Reddy D, Sumbry A, *et al.* Oral sampling and human papillomavirus genotyping in HIV-infected patients. *J Oral Pathol Med* 2012;**41**:288–91. doi:10.1111/j.1600-0714.2011.01093.x

w2 Kreimer AR, Pierce Campbell CM, Lin H-Y, *et al.* Incidence and clearance of oral human papillomavirus infection in men: the HIM cohort study. *The Lancet* 2013;**382**:877–87. doi:10.1016/S0140-6736(13)60809-0

w3 Bouvard V, Baan R, Straif K, *et al.* A review of human carcinogens--Part B: biological agents. *Lancet Oncol* 2009;**10**:321–2.

w4 Videla S, Darwich L, Cañadas M-P, *et al.* Natural history of human papillomavirus infections involving anal, penile, and oral sites among HIV-positive men. *Sex Transm Dis* 2013;**40**:3–10. doi:10.1097/OLQ.0b013e31827e87bd

w5 Parisi SG, Cruciani M, Scaggiante R, *et al.* Anal and oral human papillomavirus (HPV) infection in HIV-infected subjects in northern Italy: a longitudinal cohort study among men who have sex with men. *BMC Infect Dis* 2011;**11**:150. doi:10.1186/1471-2334-11-150

w6 Edelstein ZR, Schwartz SM, Hawes S, *et al.* Rates and determinants of oral human papillomavirus infection in young men. *Sex Transm Dis* 2012;**39**:860–7. doi:10.1097/OLQ.0b013e318269d098

w7 Herrero R, Quint W, Hildesheim A, *et al.* Reduced prevalence of oral human papillomavirus (HPV) 4 years after bivalent HPV vaccination in a randomized clinical trial in Costa Rica. *PLoS ONE* 2013;**8**:e68329. doi:10.1371/journal.pone.0068329
