# Supplementary material for: Oral human papillomavirus (HPV) infection in men who have sex with men: prevalence and lack of anogenital concordance
Source: Sex Transm Infect. 2015 Apr 17;91(4):284–6. doi: 10.1136/sextrans-2014-051955 (PMC4453633; doi:10.1136/sextrans-2014-051955)
Supplement: Web table 1 [file sextrans-2014-051955-s3.pdf]

Supplementary table 1. Selected participant characteristics of 151 HIV-negative MSM attending a sexual health clinic

	Total		Oral HPV not detected		Oral HPV detected	
	n	%	n	%	n	%
Age						
18-20	5	3.3	4	3.1	1	4.8
21-25	37	24.5	32	24.6	5	23.8
26-30	38	25.2	34	26.2	4	19.0
31-35	39	25.8	35	26.9	4	19.0
36-40	32	21.2	25	19.2	7	33.3
Ethnic group						
White	113	74.8	97	74.6	16	76.2
Black	20	13.2	17	13.1	3	14.3
Asian & SE Asian	16	10.6	14	10.8	2	9.5
Missing	2	1.3	2	1.5	0	0.0
Born in the UK						
No	75	49.7	63	48.5	12	57.1
Yes	75	49.7	66	50.8	9	42.9
Missing	1	0.7	1	0.8	0	0.0
Currently smoke						
No	110	72.8	94	72.3	16	76.2
Yes	40	26.5	35	26.9	5	23.8
Missing	1	0.7	1	0.8	0	0.0
At risk drinking (AUDIT-C)						
No	43	28.5	37	28.5	6	28.6
Yes	105	69.5	91	70.0	14	66.7
Missing	3	2.0	2	1.5	1	4.8
Currently employed						
No	29	19.2	24	18.5	5	23.8
Yes	121	80.1	105	80.8	16	76.2
Missing	1	0.7	1	0.8	0	0.0
Years of education after the age of 16						
None	4	2.6	2	1.5	2	9.5
Up to 2 years	18	11.9	13	10.0	5	23.8
3 years or more	109	72.2	97	74.6	12	57.1
Still in education	19	12.6	17	13.1	2	9.5
Missing	1	0.7	1	0.8	0	0.0
Number of lifetime partners (oral and anal)						
<=30	57	37.7	47	36.2	10	47.6
>30	93	61.6	82	63.1	11	52.4
Missing	1	0.7	1	0.8	0	0.0
Age at first oral sex with man						
Up to 15 years	34	22.5	30	23.1	4	19.0
16-20 years	82	54.3	70	53.8	12	57.1
21-25 years	30	19.9	25	19.2	5	23.8
26-39 years	3	2.0	3	2.3	0	0.0
Missing	2	1.3	2	1.5	0	0.0
Oral sex with a man in the last 3 months						
No	5	3.3	4	3.1	1	4.8
Yes	145	96.0	125	96.2	20	95.2
Missing	1	0.7	1	0.8	0	0.0
Ever had oral sex with a woman						
No	72	47.7	63	48.5	9	42.9
Yes	50	33.1	42	32.3	8	38.1
Missing	29	19.2	25	19.2	4	19.0
Ever had a warts diagnosis						
No	100	66.2	88	67.7	12	57.1
Yes	43	28.5	36	27.7	7	33.3
Don't know	6	4.0	5	3.8	1	4.8
Missing	2	6.67	1	0.8	1	4.8

Note: Formal risk factor analyses were not performed due to low numbers
