# Supplementary material for: Oral human papillomavirus (HPV) infection in men who have sex with men: prevalence and lack of anogenital concordance
Source: Sex Transm Infect. 2015 Apr 17;91(4):284–6. doi: 10.1136/sextrans-2014-051955 (PMC4453633; doi:10.1136/sextrans-2014-051955)
Supplement: Web table 2 [file sextrans-2014-051955-s4.pdf]

Supplementary table 2. HPV type distribution at oral and anogenital sites in 21 MSM positive for any HPV in oral cavity.

Oral HPV type(s)	Anogenital HPV type(s)
6	11,68
6	-
6,18	-
16	-
33	18
45	6,11,68
51	18,68
52	HPV+
56	16
56	16,39
56	-
HPV+	6,11,18,56
HPV+	16,68
HPV+	35,51,73
HPV+	45,53
HPV+	HPV+
HPV+	HPV+
HPV+	HPV+
HPV+	-
HPV+	-
HPV+	-

Key:
– anogenital samples with no detectable HPV DNA

HPV+ HPV DNA was detected but was not HPV types 6/11/16/18/31/33/35/39/45/51/52/56/58/59/68/26/53/66/70/73/82
